# Adjuvant conditioning shapes the adaptive immune response promoting immunotolerance via NLRP3/interleukin-1

**DOI:** 10.1016/j.isci.2025.112653

**Published:** 2025-05-13

**Authors:** Thais Boccia, Weikang Pan, Victor Fattori, Rodrigo Cervantes-Diaz, Michael S. Rogers, Ivan Zanoni, Alex G. Cuenca

**Affiliations:** 1 Department of Surgery, Boston Children’s Hospital - Harvard Medical School, Boston, MA, USA; 2 Vascular Biology Program, Department of Surgery, Boston Children’s Hospital - Harvard Medical School, Boston, MA, USA; 3 Department of Medicine, Division of Immunology, Boston Children’s Hospital - Harvard Medical School, Boston, MA, USA; 4 Department of Pediatrics, Division of Immunology, Division of Gastroenterology, Boston Children’s Hospital - Harvard Medical School, Boston, MA, USA

**Keywords:** Immunology, Immune response

## Abstract

Although adjuvants typically enhance immune responses, we show that repeated alum administration—termed adjuvant conditioning (AC)—induces an immunosuppressive environment that delays allogeneic graft rejection by expanding myeloid-derived suppressor cells (MDSCs). AC-induced MDSCs suppress antigen-specific adaptive responses both *in vitro* and *in vivo*, a process dependent on NLRP3 and IL-1 signaling. Allogeneic pancreatic islets transplanted into AC-treated NLRP3^−/−^ mice are not protected, confirming the necessity of NLRP3. Moreover, AC-induced MDSCs cultured with LPS exhibit reduced pro-inflammatory and increased immunosuppressive cytokine production. Similarly, prolonged alum exposure blunts inflammatory cytokine production in human cells. Together, these findings reveal that AC establishes an immunosuppressive milieu via the NLRP3/IL-1 axis. This work suggests that targeting this pathway could promote allograft tolerance in transplant recipients.

## Highlights

• Repeated alum injections before the transplant delay allograft rejection
• Pre-treatment with alum impairs the development of the adaptive immune response
• Alum expands MDSCs through NLRP3 activation and IL-1 signaling
• NLRP3−/− MDSCs function is impaired in both *in vitro* and *in vivo* models

## Graphical abstract

**Figure:**
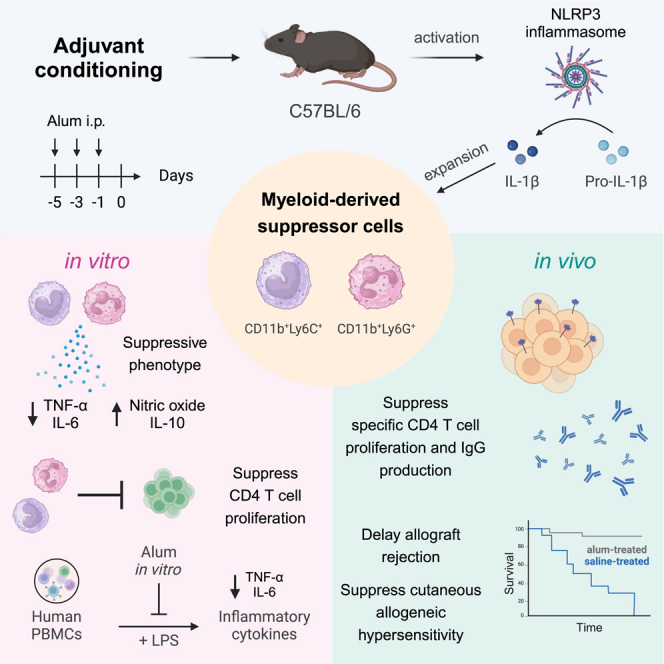

## Introduction

The utilization of adjuvants in the context of immunization potentiates the adaptive immune response driven by T and B cells. Aluminum salts (hereafter alum) are among the major adjuvants utilized in human vaccine formulations to potentiate the immune response against an antigen. Nevertheless, “non-specific” antigen-independent immunization with alum before immunization has been associated with the suppression of the adaptive immune response, with lower total and specific IgM and IgG production.^1^ Similar to alum, the systemic administration of CpG-rich DNA oligonucleotides prior to immunization has been linked to reduced T cell expansion and diminished ovalbumin-specific cytotoxic T lymphocyte (CTL) activity.^2^ Treatment of mice with an exopolysaccharide from *Bacillus subtilis* demonstrated the alleviation of graft-versus-host disease severity,^3^ highlighting the role of Toll-like receptor (TLR) activation in establishing an immunosuppressive milieu in the context of allotransplant. We previously demonstrated that, when alum is employed as an adjuvant for non-specific immunization, a strategy termed adjuvant conditioning (AC), it induced the expansion of myeloid-derived suppressor cells (MDSCs).^4^ AC-induced MDSCs suppressed T cell proliferation *in vitro* and significantly delayed the rejection of allogeneic pancreatic islets. These findings underscore the potential of AC as a pre-treatment strategy to enhance immune suppression preceding transplantation.

MDSCs represent a subset of immature myeloid cells originating from the bone marrow during systemic inflammation or cancer progression.^5^^,^^6^^,^^7^ These cells are comprised of various populations, such as monocytes and granulocytes, MDSCs, and can be detected in the peripheral blood and spleens of both mice and humans.^4^^,^^8^^,^^9^ Monocyte-MDSCs (M-MDSCs) are CD11b^+^Ly6C^high^ cells, whereas polymorphonuclear (PMN)-MDSCs exhibit CD11b^+^Ly6C^low^Ly6G^+^ markers.^10^^,^^11^ MDSCs play a significant role in promoting cancer angiogenesis and tumor growth,^12^^,^^13^ as well as contributing to anti-tumor drug resistance and metastasis.^13^^,^^14^^,^^15^^,^^16^ Moreover, MDSCs have shown promise in therapeutic applications within transplantation models in mice.^4^^,^^17^^,^^18^^,^^19^^,^^20^^,^^21^ Among the suppressive mechanisms employed by MDSCs are the release of nitric oxide and prostaglandin E_2_, potent inhibitors of T cell responses.^22^^,^^23^^,^^24^^,^^25^^,^^26^ Additionally, there is a growing recognition of the correlation between inflammasome-dependent IL-1 and the expansion, mobilization, and suppressive function of tumor-induced MDSCs.^27^^,^^28^^,^^29^^,^^30^^,^^31^^,^^32^^,^^33^^,^^34^ This association can be attributed to the dual role of this pro-inflammatory cytokine in promoting resistance to infection while also contributing to inflammation control.

As alum has been suggested to signal through the NLRP3/IL1 pathway, we explored the role of inflammasome activation in adjuvant conditioning (AC).^35^^,^^36^ First, we demonstrate that AC skews the adaptive immune response toward a Th2 phenotype in response to vaccine challenge. Next, we show that the observed effect of AC on adaptive immunity and the expansion of MDSCs is partially dependent on the NLRP3/IL1 pathway. Further, we demonstrate that AC MDSCs produce less proinflammatory cytokines, but more immunosuppressive cytokines compared to controls in response to *in vitro* stimulation with LPS, an unrelated inflammatory stimulus. In addition, we demonstrate that the previously observed protective effect of AC to alloislet rejection is abrogated in the absence of NLRP3 signaling. Further, we are also able to partially recapitulate the observed AC effects on human cells *in vitro* in response to prolonged exposure of human PBMCs to alum. These data suggest that the effect of AC on innate and adaptive immunity is at least partially dependent on NLRP3/IL-1 signaling and further that AC may induce an immunosuppressive state that can therapeutically manipulate adaptive immunity in transplant patients.

## Results

### Adjuvant conditioning shapes the adaptive immune response *in vivo*

**Figure 1:**
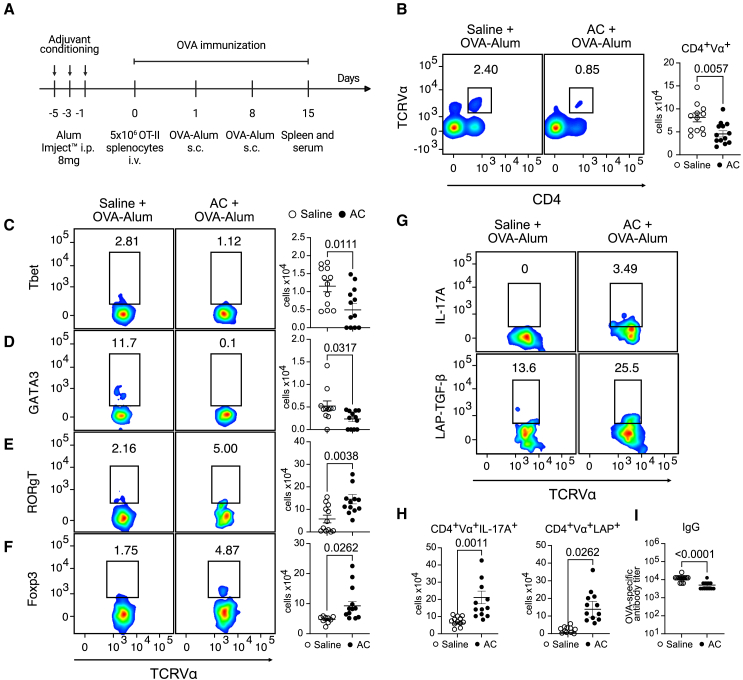
Adjuvant conditioning (AC) shapes the adaptive immune response *in vivo* (A) Experimental design. 8-10-week-old male or female C57BL/6 mice were injected intraperitoneally (i.p.) with either Alum Imject (8mg in 200 μL) or saline three times, every other day. 24 h after the last injection, mice were adoptively transferred with 5 × 10^6^ OT-II splenocytes intravenously, and 24 h after the transfer, mice were immunized subcutaneously with 10 μg OVA adsorbed in 1.5mg of Alum Imject, in a homologous prime (day 1) and boost (day 8) protocol. Seven days after the boost (day 15), mice were euthanized, and spleens were analyzed for OVA-specific Th1, Th2, Th17, and Treg total numbers. (B) Flow cytometry plots and total number of OVA-specific T cells, evidenced by the expression of CD4^+^Vα^+^ in splenocytes from mice treated with either saline or alum before the immunization. Flow cytometry plots and total number of OVA-specific Th1 (C), Th2 (D), Th17 (E), and Tregs (F), evidenced by the expression of CD4^+^Vα^+^Tbet^+^, CD4^+^Vα^+^GATA3^+^, CD4^+^Vα^+^RORγT^+^, and CD4^+^Vα^+^Foxp3^+^ in splenocytes from mice treated with either saline or alum before the immunization. (G) Flow cytometry plots and total number (H) of OVA-specific Th17 and Tregs, evidenced by the expression of CD4^+^Vα^+^IL-17A and CD4^+^Vα^+^LAP^+^ in splenocytes treated with either saline or alum before the immunization and further cultured with OVAp (2 μg/mL) *in vitro* for 48h in the presence of brefeldin A. (I) OVA-specific IgG titer in serum from mice treated with either saline or alum before the immunization. Data shown represent three or more experiments and are expressed as mean ± SEM; Student’s t test was used for analysis; *p* values are indicated in each graph. All statistical analyses were performed using GraphPad Prism. See also Figure S1.

As we have previously shown, AC expands MDSCs, which inhibit T cell proliferation *in vitro*, as well as prolong the survival of pancreatic islet transplantation in an allogeneic setting.^4^ To assess the impact of adjuvant conditioning on the development of the adaptive immune response *in vivo*, we treated mice with the conditioning protocol, which involves three subsequent injections of alum i.p., every other day, and immunized the mice with alum-adsorbed ovalbumin (OVA) after OT-II cells adoptive transfer (design experiment in Figure 1A). The number of OVA-specific CD4 T cells was increased in immunized mice compared to mice that received AC and immunization (Figures 1B and S1B). Additionally, Th1-skewed CD4^+^Vα^+^Tbet^+^ cells as well as Th2-skewed CD4^+^Vα^+^GATA3^+^ cells were significantly decreased in AC-immunized, compared to Alum-OVA-immunized, mice (Figures 1C and 1D). This was also accompanied by a subtle less production of OVA-specific IgG in AC-treated mice (Figure 1I).

Of note, AC-immunized mice exhibited enhanced differentiation of Th17 cells and regulatory T cells (Tregs) (CD4^+^Vα^+^RORγT^+^ and CD4^+^Vα^+^Foxp3^+^, respectively), compared to Alum-OVA-immunized mice (Figures 1E and 1F). This differentiation pattern was further confirmed when splenocytes were restimulated *in vitro* with OVA, as AC-immunized mice displayed elevated production of interleukin-17A (IL-17A) and transforming growth factor β (TGF-β) (CD4^+^Vα^+^IL-17A^+^ and CD4^+^Vα^+^LAP^+^) (Figures 1G and 1H). These results demonstrate that AC treatment dampens the adaptive immune response.

### NLRP3 activation is involved in the effects of adjuvant conditioning on the adaptive immune response *in* vivo

Alum is a widely employed adjuvant in immunization protocols,^37^ and its activity has been shown to involve NLRP3 activation,^35^^,^^36^ however, this is a controversial topic, since there are reports of specific antibody production in the absence of inflammasome activation.^38^^,^^39^ Indeed, in our model, we confirmed that NLRP3-deficient mice exhibited reduced the expansion of OVA-specific CD4 T cells and lower levels of OVA-specific IgG compared to WT mice when immunized with OVA in the presence of alum (Figures S2B and S2C), as previously shown.^35^^,^^40^

**Figure 2:**
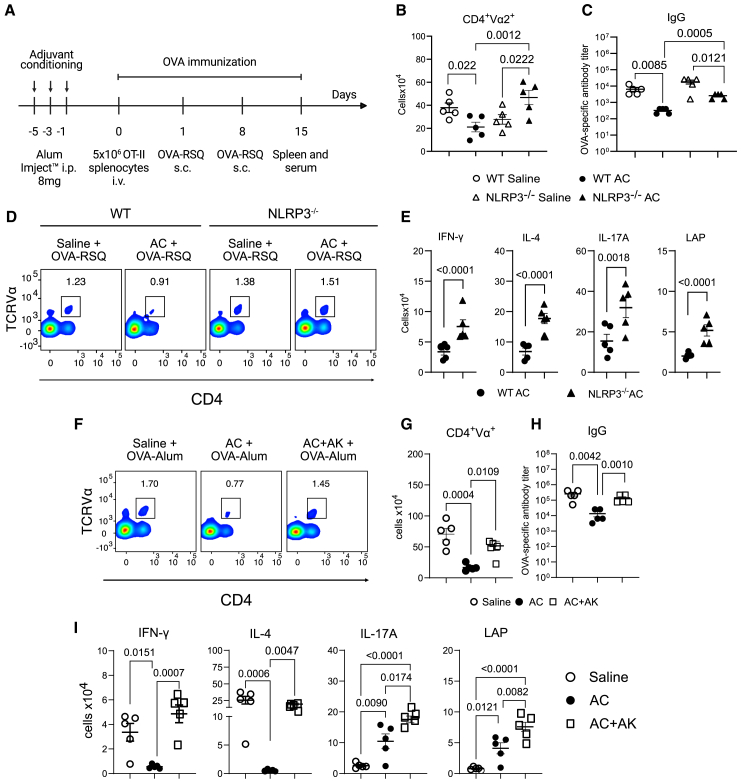
NLRP3 activation and IL-1 signaling are required for the effects of adjuvant conditioning in the adaptive immune response (A) Experimental design. 8-10-week-old male or female WT or NLRP3^−/−^ mice were injected intraperitoneally (i.p.) with either Alum Imject (8mg in 200μL) or saline three times, every other day. 24 h after the last injection, mice were adoptively transferred with 5 × 10^6^ OT-II splenocytes intravenously, and 24 h after the transfer, mice were immunized with 10 μg OVA adsorbed in Resiquimod (R848) (OVA-RSQ) (50μg), in a homologous prime (day 1) and boost (day 8) protocol. Seven days after the boost (day 15), mice were euthanized, and spleens were analyzed for OVA-specific Th1, Th2, Th17, and Treg total numbers. (B) Total number of OVA-specific T cells, evidenced by the expression of CD4^+^Vα^+^ in splenocytes from mice treated with either saline or alum before the immunization. (C) OVA-specific IgG titer in serum from mice treated with either saline or alum before the immunization. (D) Flow cytometry plots of OVA-specific T cells, evidenced by the expression of CD4^+^Vα^+^ in splenocytes from mice treated with either saline or alum before the immunization. (E) Total number of OVA-specific Th1, Th2, Th17 and Tregs, evidenced by the expression of CD4^+^Vα^+^IFN-γ^+^, CD4^+^Vα^+^IL-4^+^, CD4^+^Vα^+^IL-17A^+^, and CD4^+^Vα^+^LAP^+^, respectively in splenocytes treated with either saline or alum before the immunization and further cultured with OVAp (2 μg/mL) *in vitro* for 48h in the presence of brefeldin A. (F) Flow cytometry plots and total number (G) of OVA-specific T cells, evidenced by the expression of CD4^+^Vα^+^ in splenocytes from mice treated with either saline, alum, or alum ^+^ Anakinra (30 mg/kg) (AC^+^AK) before the immunization. (H) OVA-specific IgG titer in serum from mice treated with either saline, alum or alum + Anakinra (30 mg/kg) (AC + AK) before the immunization. (I) Total number of OVA-specific Th1, Th2, Th17 and Tregs, evidenced by the expression of CD4^+^Vα^+^IFN-γ^+^, CD4^+^Vα^+^IL-4^+^, CD4^+^Vα^+^IL-17A^+^ and CD4^+^Vα^+^LAP^+^ in splenocytes treated with either saline, alum or alum + Anakinra (30 mg/kg) (AC + AK) before the immunization and further cultured with OVAp (2 μg/mL) *in vitro* for 48h in the presence of brefeldin A. Data shown represent three or more experiments, and are expressed as mean ± SEM; Student’s t test was used for analysis; *p* values are indicated in the graphs. All statistical analyses were performed using GraphPad Prism. See also Figure S2.

To assess whether the effects of AC involve NLRP3 activation, we performed AC with alum and immunized mice with OVA and utilized resiquimod (R848) as an adjuvant^41^ (Figure 2A) to avoid NLRP3 involvement in the development of the adaptive immune response. We found that AC dampened CD4 T cell responses to OVA, as well as OVA-specific IgG and IgE antibody levels, also in the presence of resiquimod (Figures 2B–2D and S2C). The effects on AC-treated mice were lost or significantly decreased in the absence of NLRP3 (Figures 2B–2D and S2C). We also observed that AC decreased Th1 and Th2 responses under these experimental conditions (Figures 2E and S2D). Furthermore, AC followed by the immunization of mice deficient in NLRP3 heightened differentiation not only of Th1 and Th2 cells but also of Th17 cells and Tregs (Figures 2E and S2D). Overall, these data demonstrate an important role for the NLRP3 inflammasome in driving the immunosuppression that follows AC.

### NLRP3-dependent IL-1 signaling is involved in the effects of adjuvant conditioning on the adaptive immune response *in vivo*

Although extensive literature exists regarding the inflammatory effects of IL-1, recent studies have shown its capacity to stimulate the expansion of immunosuppressive cells such as MDSCs and Tregs, consequently promoting tumor growth.^29^^,^^31^^,^^42^^,^^43^ Notably, we found that NLRP3 deficiency impairs the production of both IL-1α and IL-1β following AC (Figure S2A). To determine whether the AC-driven immunosuppression involves IL-1 signaling, we used Anakinra to block IL-1 signaling during AC, followed by the adoptive transfer of OT-II splenocytes and alum-OVA immunization. Our findings indicate that IL-1 blockade restored the proliferation of OVA-specific CD4 T cells (Figures 2F and 2G), facilitated Th1 and Th2 differentiation (Figures 2I and S2E), and restored OVA-specific IgG and IgE production (Figures 2H and S2F). Intriguingly, the elevation in Th17 and Treg differentiation persisted (Figures 2I and S2E), suggesting that the Th17/Treg phenotype induced by the conditioning is independent of IL-1 signaling during AC, indicating other effects that AC may induce effects that do not involve NLRP3 activation and IL-1.

### Adjuvant conditioning induces the reprogramming of myeloid cells to an immunosuppression phenotype

**Figure 3:**
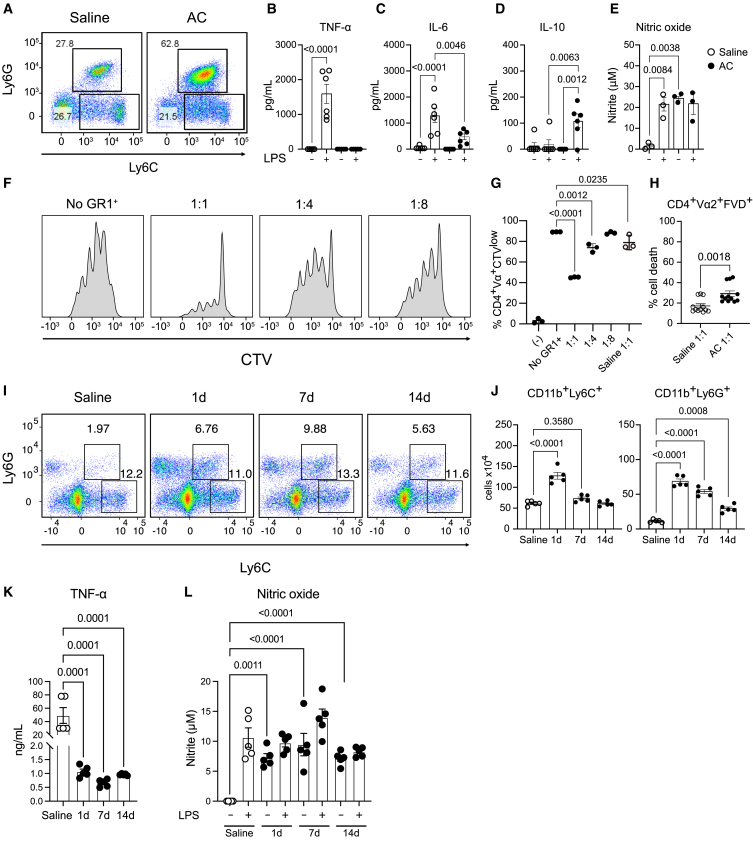
Adjuvant conditioning induces the reprogramming of myeloid cells to an immunosuppressed phenotype (A) Flow cytometry plots of CD11b^+^Ly6C^+^ and CD11b^+^Ly6G^+^ cells in the spleen 24h after saline or alum injections. (B) TNF-α, IL-6 (C), IL-10 (D), and nitric oxide (E) production from CD11b^+^GR1^+^ cells isolated from saline or AC-treated mice cultured with either medium or LPS (200 ng/mL) for 24h. (F) Histograms of CD4^+^Vα^+^ cells in a suppression assay using isolated CD11b^+^GR1^+^ cells. Isolated MDSCs from spleens from AC-treated mice were cultured with OVAp (1 μg/mL), WT splenocytes (1 × 10^5^) and isolated OT-II naive CD4 T cells (2 × 10^5^) in the ratios (MDSC: T cell) indicated in the graph. (G) Percentage of CD4^+^Vα^+^CTV^low^ cells in a suppression assay using isolated CD11b^+^GR1^+^ cells and OT-II CD4 T cells. (H) Percentage of CD4^+^Vα^+^FVD^+^ cells in a suppression assay using isolated CD11b^+^GR1^+^ cells and OT-II CD4 T cells. (I) Flow cytometry plots of CD11b^+^Ly6C^+^ and CD11b^+^Ly6G^+^ cells in the spleen 1 day, 7 days, and 14 days after saline or alum injections. (J) Total count of CD11b^+^Ly6C^+^ and CD11b^+^Ly6G^+^ cells in the spleen 1 day, 7 days, and 14 days after saline or alum injections. (K) TNF-α and nitric oxide (L) production from CD11b^+^GR1^+^ cells isolated 1 day, 7 days, and 14 days after saline or alum injections were cultured with either medium or LPS (200 ng/mL) for 24h. Data shown represent three or more experiments and are expressed as mean ± SEM; Student’s t test was used for analysis; *p* values are indicated in each graph. All statistical analyses were performed using GraphPad Prism. See also Figure S3.

In agreement with our previous findings that showed the capacity of AC to expand MDSCs, AC led to an increase in total splenocytes and spleen weight (Figures S3A and S3B), and to the expansion of CD11b^+^Ly6C^+^ and CD11b^+^Ly6G^+^ cells (Figures 3A, S3C, and S3D).^4^ To test the immunosuppressive activity of MDSCs differentiated upon AC treatment, we isolated splenic CD11b^+^GR1^+^ cells from saline or AC-treated mice were isolated and treated them with LPS (200 ng/mL) *in vitro*. CD11b^+^GR1^+^ cells from conditioned mice produced reduced levels of TNF-α and IL-6 compared to the cells isolated from saline-treated mice (Figures 3B and 3C). Additionally, these cells showed elevated levels of IL-10 and nitric oxide (Figures 3D and 3E) compared to their saline-treated counterparts, indicating an immature neutrophil/monocyte suppressor programming.^29^^,^^44^ In functional assays, AC-induced splenic CD11b^+^GR1^+^ cells demonstrated potent suppression of CD4 T cell proliferation, with efficacy extending up to a 1:4 MDSC:T cell ratio (Figures 3F and 3G) and induced increased CD4 T cell death when compared to saline CD11b^+^GR1^+^ at a 1:1 ratio (Figure 3H). It is important to note that CD11b^+^GR1^+^ isolated from saline treated mice are able to somewhat suppress T cell proliferation at 1:1 ratio (Figure 3G) but are not as potent compared to the suppression from cells observed from MDSCs isolated from AC-treated mice.

Furthermore, the anti-inflammatory effect persisted up to 14 days following conditioning. This is evidenced by sustained levels of CD11b^+^Ly6C^+^ and CD11b^+^Ly6G^+^ cells (Figures 3I and 3J), with a gradual increase in PD-L1, and sustained IDO expression (Figures S3F–S3H). When isolated, CD11b^+^GR1^+^ cells exhibit low TNF-α production upon LPS treatment *in vitro* (Figure 3K), while nitric oxide production was present regardless of LPS stimulation (Figure 3L). These results suggest that AC leads to a long-lasting reprogramming of CD11b^+^GR1^+^ cells that acquire an immunosuppressive phenotype.

### Adjuvant conditioning-induced myeloid-derived suppressor cell expansion and suppressor function require NLRP3 activation and interleukin-1 signaling

**Figure 4:**
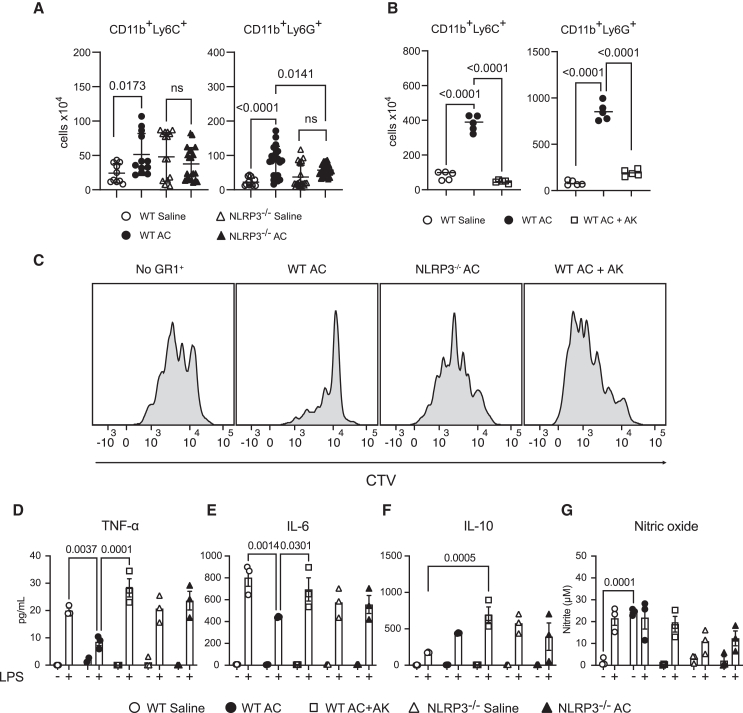
AC-induced MDSCs expansion and suppressor function requires NLRP3 activation and IL-1 signaling (A) Total number of CD11b^+^Ly6C^+^ and CD11b^+^Ly6G cells in the spleen 24h after saline or alum injections in WT and NLRP3 deficient mice. (B) Total number of CD11b^+^Ly6C^+^ and CD11b^+^Ly6G cells in the spleen 24h after saline, alum, or alum+Anakinra (30 mg/kg) (AC + AK) injections. (C) Histograms of CD4^+^Vα^+^ cells in a suppression assay using isolated CD11b^+^GR1^+^ from WT and NLRP3 deficient mice treated with saline, alum, or alum+Anakinra (30 mg/kg) (AC + AK), and percentage of CD4^+^Vα^+^CTV^low^ cells. (D) TNF-α, IL-6 (E), IL-10 (F), and nitric oxide (G) production from CD11b^+^GR1^+^ cells isolated from WT or NLRP3^−/−^ mice injected either with saline, alum, or alum+Anakinra (30 mg/kg) (AC + AK), cultured with either medium or LPS (200 ng/mL) for 24h. Data shown represent three or more experiments, and are expressed as mean ± SEM; Student’s t test was used for analysis; *p* values are indicated in each graph. All statistical analyses were performed using GraphPad Prism.

Since there are reports of NLRP3 and IL-1 involvement in MDSC expansion and suppressor function in tumor models,^29^ we next assessed the role of the NLRP3/IL-1 axis on AC-induced MDSC expansion and suppressor function. AC-treated NLRP3^−/−^ mice revealed a notable decrease in the expansion of CD11b^+^Ly6C^+^ and CD11b^+^Ly6G^+^ populations compared to their wild-type counterparts (Figure 4A). Additionally, we observed that the immunosuppressive function of AC-induced CD11b^+^GR1^+^ cells was impaired in the absence of NLRP3 (Figures 4C and S3J).

To further explore the requirement for IL-1 signaling downstream of NLRP3 activation for MDSC expansion and suppressor function, we administered Anakinra during the conditioning protocol. We observed that the expansion of both CD11b^+^Ly6C^+^ and CD11b^+^Ly6G^+^ populations was reduced, accompanied by impaired immunosuppressive function when compared to AC-induced MDSCs derived from WT mice (Figures 4B, 4C, and S3L–S3M). This suggests that NLRP3 activation and subsequent IL-1 signaling during AC are crucial for the expansion and suppressor function of MDSCs, as previously shown for tumor-induced MDSCs.^29^ Furthermore, we found no evidence of the anti-inflammatory phenotype in AC-treated NLRP3^−/−^ CD11b^+^GR1^+^ cells, or in the presence of IL-1 signaling blockade. Instead, we observed increased production of TNF-α and IL-6, alongside diminished nitric oxide release in NLRP3^−/−^ CD11b^+^GR1^+^ cells treated with LPS (Figures 4D–4G), further suggesting a possible role of NLRP3 signaling in the reprogramming of CD11b^+^GR1^+^ cells to an immunosuppressive phenotype.

### Adjuvant conditioning-induced myeloid-derived suppressor cells inhibit the adaptive immune response *in vivo* via NLRP3 activation and interleukin-1 signaling

**Figure 5:**
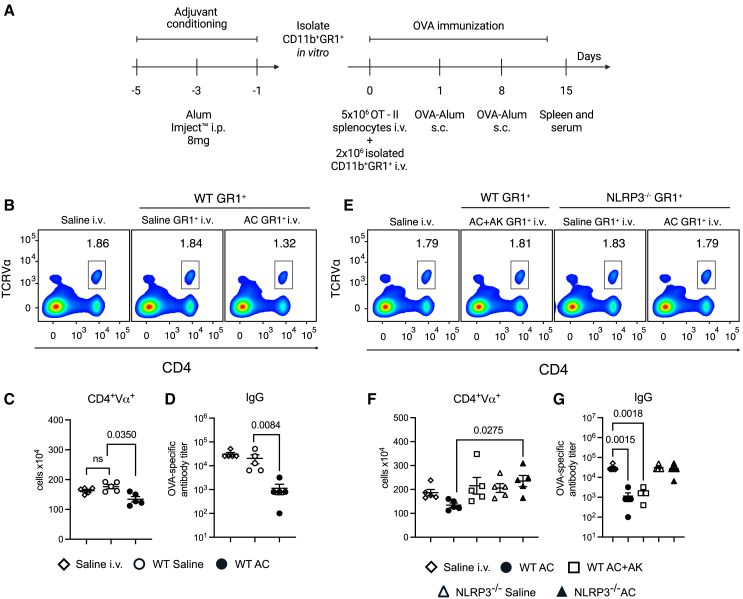
AC-induced MDSC inhibition of the adaptive immune response *in vivo* requires NLRP3 activation and IL-1 signaling (A) Experimental design. Briefly, 2 × 10^6^ CD11b^+^GR1^+^ cells isolated from either AC, alum + Anakinra (30 mg/kg) (AC + AK) or saline-injected WT or NLRP3^−/−^ mice were adoptively transferred to C57BL/6 mice before the immunization protocol. (B) Flow cytometry plots and total number of OVA-specific T cells (C), evidenced by the expression of CD4^+^Vα^+^ in splenocytes from mice adoptively transferred with CD11b^+^GR1^+^ cells isolated from either AC or saline-injected C57BL/6 before the immunization protocol. (D) OVA-specific IgG titer in serum from mice adoptively transferred with CD11b^+^GR1^+^ cells isolated from either AC or saline-injected WT mice before the immunization protocol. (E) Flow cytometry plots and total number of OVA-specific T cells (F), evidenced by the expression of CD4^+^Vα^+^ in splenocytes from mice adoptively transferred with CD11b^+^GR1^+^ cells isolated from either alum+Anakinra (30 mg/kg) (AC + AK) or saline-injected WT or NLRP3^−/−^ mice before the immunization protocol. (G) OVA-specific IgG titer in serum from mice adoptively transferred with CD11b^+^GR1^+^ cells isolated from either alum+Anakinra (30 mg/kg) (AC^+^AK) or saline-injected WT or NLRP3^−/−^ mice before the immunization protocol. Data shown represent three or more experiments and are expressed as mean ± SEM; Student’s t test was used for analysis; *p* values are indicated in the graphs. All statistical analyses were performed using GraphPad Prism.

To investigate the impact of AC-induced MDSCs on shaping the adaptive immune response *in vivo*, we adoptively transferred CD11b^+^GR1^+^ cells isolated from AC or saline-treated mice into C57BL/6 recipients and mice immunized with Alum-OVA (Figure 5A). Mice that received WT AC-induced MDSCs exhibited impaired expansion of OVA-specific CD4 T cells compared to those receiving CD11b^+^GR1^+^ cells from WT saline-injected mice (Figures 5B and 5C). Moreover, recipients of WT AC-induced MDSCs showed diminished levels of OVA-specific IgG production (Figure 5D), demonstrating that WT CD11b^+^GR1^+^ cells induced during AC exposure also suppress the adaptive immune response *in vivo*.

To explore the involvement of the NLRP3/IL-1 axis in MDSCs' suppressor function *in vivo*, we adoptively transferred AC or saline-induced CD11b^+^GR1^+^ cells isolated from NLRP3-deficient mice, or WT mice treated with Anakinra during the AC-treatment. Mice receiving NLRP3-deficient AC or saline-induced MDSCs demonstrated comparable expansion of OVA-specific CD4 T cells in both percentage and absolute numbers to control immunized mice, and similar results were obtained in mice receiving CD11b^+^GR1^+^ cells from Anakinra-treated donors (Figures 5E and 5F). Furthermore, OVA-specific IgG production was restored in mice that received NLRP3-deficient AC or saline-induced CD11b^+^GR1^+^ cells, but not in those those receiving cells from Anakinra-treated WT donors (Figure 5G). Overall, these data underscore the role of the NLRP3/IL-1 axis in the suppressive function of AC-induced MDSCs *in vivo*.

### Adjuvant conditioning-induced *in vivo* immunosuppressive phenotype requires myeloid-derived suppressor cells activation

**Figure 6:**
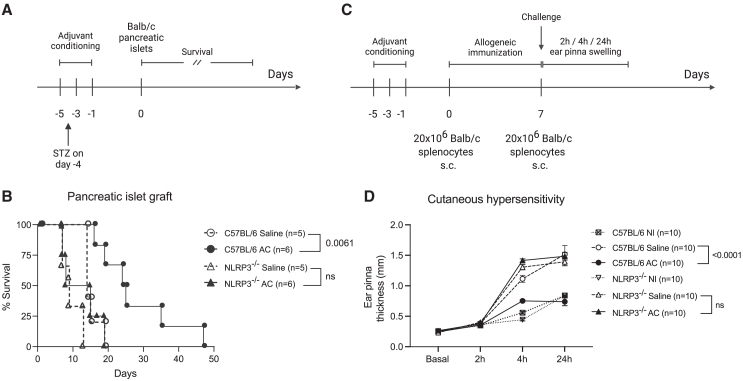
Adjuvant conditioning promotes allogeneic tolerance dependent on NLRP3 (A) Allogeneic pancreatic islet transplantation experimental design. Briefly, alum or saline-treated WT or NLRP3^−/−^ mice were treated with streptozotocin (STZ) 4 days before the transplantation with Balb/c isolated pancreatic islets. Survival of the graft was assessed daily by checking blood glucose levels. (B) Survival curve of alum or saline-treated WT or NLRP3^−/−^ mice that underwent allogeneic pancreatic islet transplantation. WT saline (*n* = 5), AC (*n* = 6), NLRP3^−/−^ saline (*n* = 5), AC (*n* = 6). Graft survival significance was assessed by Kaplan-Meier/Mantel-Cox log rank test. (C) Cutaneous hypersensitivity experimental design. Briefly, alum or saline-treated WT or NLRP3^−/−^ mice were subcutaneously immunized with 20 × 10^6^ Balb/c splenocytes on the neck. Seven days later, mice were challenged with 20 × 10^6^ Balb/c splenocytes injected subcutaneously into the base of the ear, as described by Zecher et al. (2009).^45^ (D) Cutaneous allogeneic response was assessed by measuring ear pinna swelling (mm) at 2-, 4- and 24-h post-challenge. *p*-values show the comparison between ear swelling at 24-h post-challenge. *n* = 10 for each group. Data shown represent three or more experiments and are expressed as mean ± SEM; Student’s t test was used for analysis; *p* values are indicated in each graph. All statistical analyses were performed using GraphPad Prism.

We previously demonstrated that adjuvant conditioning prolongs the survival of allogeneic pancreatic islet transplants through the expansion of MDSCs.^4^ Since we the data above suggests that AC-induced MDSCs expansion and suppression function are dependent on NLRP3, we explored whether this effect extends to allogeneic responses *in vivo* and performed allogeneic islet transplantation in WT and NLRP3^−/−^ saline or AC-treated mice (Figure 6A). Our findings revealed that AC significantly delays allogeneic islet rejection in WT but not in NLRP3-deficient mice (Figure 6B), underscoring the crucial role of NLRP3 in promoting the observed protective effect of AC *in vivo*.

We further investigated the impact of adjuvant conditioning on the allogeneic response using an additional model. The cutaneous allogeneic response was assessed by measuring ear pinna swelling up to 24 h post-challenge (Figure 6C). Conditioned WT mice exhibited reduced ear swelling compared to saline-treated controls (Figure 6D), confirming the suppressive effect of the conditioning protocol on the allogeneic response. Consistent with our allogeneic islet transplantation experiments, conditioned NLRP3^−/−^ mice failed to suppress the allogeneic response, showing similar levels of ear swelling as saline-treated controls (Figure 6D). Collectively, the data strongly suggest the critical role of NLRP3 in the creation of an immunosuppressive milieu that is capable of regulating allogeneic responses *in vivo* following AC.

### Alum stimulation induces an immunosuppressive phenotype in human PBMCs *in vitro*

**Figure 7:**
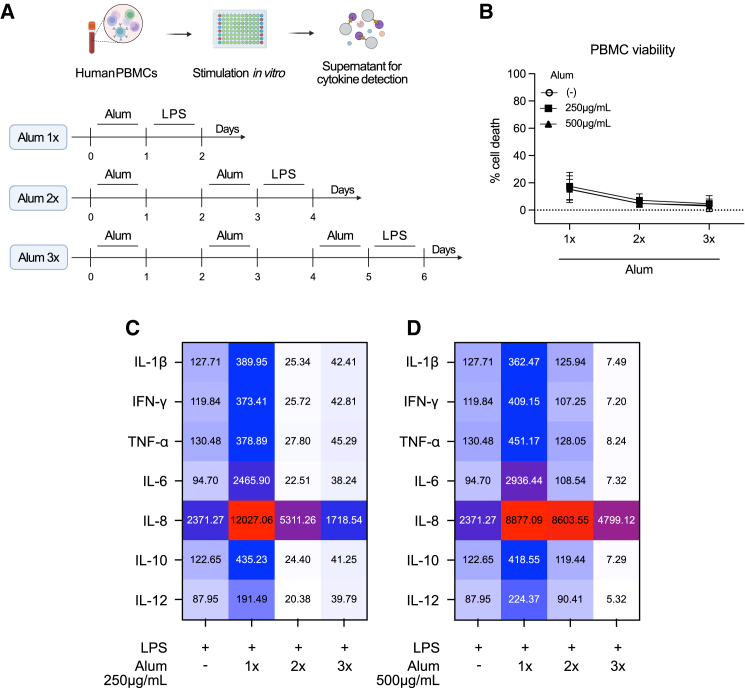
Alum stimulation induces immunosuppression in human PBMCs *in vitro* (A) Experimental design. Briefly, 3 × 10^5^ total PBMCs from five different healthy donors were stimulated with alum (250 μg/mL and 500 μg/mL) once, twice or three times before stimulation with LPS (200 ng/mL) for 24h in round bottom 96-well plates, in complete RPMI (described in STAR Methods). At the designated times, the supernatant was collected for cytokine measurement using LEGENDplex Human Inflammation Panel 1. (B) Viability of PBMCs stimulated with alum (250 μg/mL and 500 μg/mL) once, twice, or three times before stimulation with LPS (200 ng/mL), measured by LDH release. (C) IL-1β, IFN-γ TNF-α, IL-6, IL-8, IL-10, and IL-12 production by PBMCs stimulated with alum (250 μg/mL) once, twice or three times before stimulation with LPS (200 ng/mL). (D) IL-1β, IFN-γ TNF-α, IL-6, IL-8, IL-10, and IL-12 production by PBMCs stimulated with alum (500 μg/mL) once, twice or three times before stimulation with LPS (200 ng/mL). Numbers in squares show the mean of the 5 donor cells for each cytokine in pg/mL. Figure created with BioRender.

Finally, we investigated whether adjuvant conditioning-induced immunosuppression also occurs in humans; we adapted an innate training protocol for *in vitro* studies.^46^^,^^47^ Human PBMCs were stimulated with alum (once, twice, or three times) before LPS stimulation (Figure 7A) in an attempt to recapitulate adjuvant conditioning. The supernatant was collected for viability assays and cytokine detection. We observed that a single alum stimulation induced an increase in nearly all the inflammatory cytokines tested after LPS stimulation. When cells were treated with multiple doses of alum, the secretion of the proinflammatory cytokines, and especially IL-12, TNF-α, IL-8, and IL-6, declined (Figure 7C), and this effect depended on the dose of alum (Figure 7D). Viability assays confirmed sufficient cell survival 24 h after each alum stimulation; however, there was a slight increase in monocyte cell death, compared to T and B cells (Figures 7B, S4A, and S4B), suggesting either an active immunosuppressive phenotype or that PBMCs are developing resistance to LPS signaling. These findings demonstrate that AC drives immunosuppression, or maybe a resistance to inflammatory signaling both in mouse and human cells.

## Discussion

Currently, several strategies are employed to induce immunosuppression in patients with autoimmune diseases or undergoing transplantation, including various clinical trials testing new drugs and cell based therapy protocols.^48^^,^^49^^,^^50^ Exploring the potential of FDA-approved adjuvants such as alum represents a novel approach to these protocols. These substances, traditionally known for enhancing immune responses to foreign antigens, could also be leveraged to expand immunosuppressive cell populations, potentially aiding in the induction of tolerance. Our group has previously demonstrated the potential of aluminum hydroxide salts in the protection of mice from neonatal sepsis, inducing emergency myelopoiesis,^51^ and prolonging allogeneic graft survival.^4^ Herein, we demonstrate that adjuvant conditioning, in an NLRP3 dependent manner, expands MDSCs, which effectively suppresses antigen-specific adaptive immune response both *in vitro* and *in vivo,* supporting the concept that AC induces immunosuppression and facilitates tolerance.

The effects of adjuvant conditioning before immunization and/or infections have been studied by other groups; however, the mechanism by which each adjuvant exerts that effect is different. In a protocol similar to what we describe here as adjuvant conditioning, EPS, an exopolysaccharide derived from *Bacillus subtilis*, has demonstrated protective effects in various contexts: preventing allergic eosinophilia,^52^^,^^53^^,^^54^ combating infections caused by *C. rodentium*^55^ and *S. aureus*,^56^ and mitigating severe GVHD.^3^ EPS exerts its immunosuppressive actions through a mechanism involving TLR4-dependent IDO expression in dendritic cells.^57^^,^^58^ Similarly, CpG, another TLR ligand, when administered during conditioning prior to immunization, reduces total IgG titers^1^^,^^2^ and suppresses CD8 T cell cytotoxic responses via an IDO-dependent mechanism,^2^ highlighting a consistent pattern of TLR-induced immunosuppressive mechanisms. Furthermore, although controversial, some studies have demonstrated that aluminum hydroxide salts have been noted for their ability to suppress antibody production, either alone or in combination with CpG.^1^^,^^2^

Previous studies have shown consistent findings regarding inflammasome activation occurring prior to immunization or during infections. Inflammasomes are multi-protein complexes that assemble upon the cytosolic detection of microbial patterns or harmful stimuli, resulting in the activation of caspase-1, pyroptotic cell death, and the release of IL-1β and IL-18. These processes play a critical role in the control of bacterial and parasitic infections.^59^^,^^60^^,^^61^ Despite their association with increased inflammation, inflammasome activation has been reported to exert anti-inflammatory effects as well. Notably, NLRP3 activation has been linked to immunosuppressive outcomes such as reduced CD8 T cell responses against tumors^62^^,^^63^^,^^64^^,^^65^ and induction of protective responses in colitis.^66^^,^^67^ Activation of caspase-1, a key effector of the inflammasome complex, has been observed to contribute to CD4 T cell depletion during HIV infection,^68^ while blocking IL-18 signaling in T cells may exacerbate disease severity in murine colitis models.^67^^,^^69^ Our findings, showing reduced expansion of specific CD4 T cells and diminished antibody production following the conditioning regimen, are consistent with these documented effects in the literature. However, our findings associated with the suppression of antigen specific IgG responses are controversial, as there have been reports both supporting these findings, as well as contradicting these results. In our study, the effect on immunoglobulin production is likely more reflective of the expansion of MDSCs, which have been shown to suppress IgG and modulate humoral responses as opposed to a direct effect of alum itself.^70^^,^^71^^,^^72^ Regardless, we certainly acknowledge this limitation in the explanation for the mechanism of humoral suppression.

NLRP3-dependent IL-1 has been implicated in the generation, expansion, and suppressor function of MDSCs in mouse tumor models.^29^^,^^30^^,^^31^^,^^33^^,^^34^^,^^65^^,^^73^^,^^74^ Our study also demonstrates a critical role of NLRP3 activation and IL-1 signaling in promoting both the expansion and suppressive function of these MDSCs *in vitro* and *in vivo*. However, the effect of suppressive effect of LPS stimulation on AC MDSCs is unexpected and suggests that NLRP3/IL1 signaling could lead to a tolerogenic response.

Overall, our findings demonstrate that AC can shape both innate immunity and subsequent adaptive immune responses through the activation of NLRP3. Taken together, these data imply that adjuvants designed to target the NLRP3/IL1 pathway can be used to produce an immunosuppressive milieu to ameliorate proinflammatory pathologies and to condition recipients prior to transplantation.

### Limitations of the study

We showed that repeated alum administration is able to suppress the adaptive immune response, and this immunosuppressive environment involves the expansion of MDSCs through an NLRP3/IL-1 dependent mechanism. We used a global NLRP3 knockout mouse strain, which allowed us to show the involvement of NLRP3 activation in the expansion and suppressor function of MDSCs by alum, but it still does not give us evidence of which cell types require inflammasome activation for the phenotype we observed. Certainly, the use of conditional inflammasome components knock out in myeloid cells would give us more insights into the mechanism of adjuvant conditioning. Finally, we understand the limitation of the *in vitro* stimulation of PBMCs in an attempt to simulate adjuvant conditioning in humans may not be the best approach to investigate possible changes to immune cells induced by alum, given that we only observed a reduction of inflammatory cytokine production, and did not investigate the transcriptional and epigenetic changes in the different cell types. However, the results we have observed in mice might not be fully recapitulated in PBMCs, given the complexity of the immune response, the inherent well known variability of cytokine production in human samples as seen in Figure S5,^75^^,^^76^^,^^77^ and the requirement for a complete organism to study this mechanism. Our study presents insight into a novel pathway promoting suppressive innate immunity and provides the platform for the additional studies mentioned above to further explore the therapeutic role of adjuvant conditioning in solid organ transplant.

## Resource availability

### Lead contact

Further information and requests for resources should be directed to and will be fulfilled by the lead contact, Alex Cuenca.

### Materials availability

This study did not generate new unique reagents.

### Data and code availability

Data acquired specifically for this study are available within the article itself and the supplemental information. Experimental protocols and additional details regarding methods employed in this study will be made available through reasonable request with the corresponding author.

## Acknowledgments

This work was supported by the American Pediatric Surgical Association Jay Grosfeld Scholar Grant, 10.13039/100014285Society of University Surgeons Junior Faculty Award, Hardy Hendren Faculty Development Fund at Boston Children’s Hospital, and the Junior Translational Investigator Service Award from the Translational Research Program at Boston Children’s Hospital.

## Author contributions

Conceptualization, A.G.C. and T.B.; methodology, T.B., V.F., R.C., and W.P.; investigation, T.B. and W.P.; writing – original draft, T.B. and A.G.C.; writing – review and editing, T.B., V.F., R.C., M.S.R., I.Z., and A.G.C.; funding acquisition, A.G.C.; resources, M.S.R., V.F., and A.G.C.; supervision, A.G.C.

## Declaration of interests

The authors declare no competing interests.

## STAR★Methods

### Key resources table

**Table:** 

REAGENT or RESOURCE	SOURCE	IDENTIFIER
**Antibodies**		

CD11b PE	Invitrogen, clone M1/70	12-0112-82
Ly6C PeCy7	BioLegend, clone HK1.4	128016
Ly6G PercP-Cy5	BioLegend, clone 1A8	127616
CD3 FITC	Invitrogen, clone 17A2	11-0032-82
CD4 PeCy7	Invitrogen, clone GK1.5	25-0041-82
PD-L1 BV421	BioLegend, clone 10F.9G2	124315
T-bet BV605	BioLegend, clone 4B10	644821
GATA3 PE	BioLegend, clone 16E10A23	653804
RORgT BV421	BioLegend, clone Q31-378	656013
Foxp3 PercP-Cy5	BioLegend, clone MF-14	320016
IFN-g PE	BioLegend, clone AN-18	505808
IL-4 BV421	BioLegend, clone 11B11	504122
IL-17-A BV605	BioLegend, clone TC11-18H10	506940
LAP PercP-Cy5	BioLegend, clone TW7-16B4	141410
TCR Va2 PE	BioLegend, clone B20.1	127806
TCR Va2 PercP-Cy5	BioLegend, clone B20.1	127808
Purified anti-IDO Antibody	BioLegend, clone 2E2/IDO1	122402
Purified anti-β-actin Antibody	BioLegend	664801
HRP Goat anti-rat IgG	BioLegend	405405
HRP Goat anti-mouse IgG	Sigma-Aldrich	A4416
Anakinra - Kineret	Sobi	NDC 66658-230-01

**Biological samples**		

Human PBMCs	Leukoreduction System Cones from Boston Children’s Hospital blood bank	

**Chemicals, peptides, and recombinant proteins**		

Alum Adjuvant	G-Biosciences	786-1215
Lysis buffer ACK	Gibco	A1049201
STZ	Sigma	S0130
Saline	Gibco	14190250
LPS	Sigma	L4391
Resiquimod (R848)	Invivogen	tlrl-r848
OVAp	Invivogen	vac-pova
OVA-V	Sigma	V1701
RPMI-1640 Medium	Corning	10-040-CV
Penicillin-Streptomycin (10000 U/mL)	Gibco	15140122
MEM Non-Essential Amino Acids Solution (100X)	Gibco	11140050
Fetal Bovine Serum	Gibco	26140079
Sodium Pyruvate (100 mM)	Gibco	11360070
2ME (2-Mercaptoethanol)	Gibco	21985023
Lymphoprep density gradient medium centrifugation	STEMCELL Technologies	07801
EDTA	Sigma-Aldrich	E9884
PBS 10x	Corning	46-013-CM
Tween 20	Sigma-Aldrich	P1379
Carbonate-Bicarbonate Buffer with Azide BioUltra, tablet	Sigma-Aldrich	08058
OPD (o-Phenylenediamine Dihydrochloride)	Sigma-Aldrich	P8287
TBS 10X, pH 7.4, DNase/RNase and protease free	Corning	46-012-CM
BSA (Bovine Serum Albumin)	Sigma-Aldrich	A2153
Sulfuric Acid, 99.999%	Sigma-Aldrich	339741

**Critical commercial assays**		

Mouse IL-1a ELISA	R&D Systems	MLA00
Mouse IL-1b ELISA	R&D Systems	MLB00C
LEGENDplex™ Mouse Inflammation Panel (13-plex) with V-bottom Plate	BioLegend	740446
LEGENDplex™ Human Inflammation Panel (13-plex) with V-bottom Plate	BioLegend	740389
CyQUANT™ LDH Cytotoxicity Assay Kit	Invitrogen	C20300
Griess Reagent Kit	Invitrogen	G7921
Fixation/Permeabilization Kit	BD Biosciences	554714
Mouse MDSC Enrichment Kit	StemCell Technologies	19762
Mouse Naïve CD4^+^ T Cell Isolation Kit	StemCell Technologies	19765
Fixable Viability Dye (FVD) eFluor™ 780	Invitrogen	65-0865-14
Zombie Violet™ Fixable Viability Kit	BioLegend	423113
CellTrace™ Violet Cell Proliferation Kit, for flow cytometry	Invitrogen	C34557
Accu-Chek Guide Meter	Accu-check	07562462001
Accu-Chek Guide Test Strips (50 count)	Accu-check	07453744119

**Experimental models: Organisms/strains**		

C57BL/6J (H-2^b^) mice	Jackson Laboratory	#000664
Balb/C (H-2^d^) mice	Jackson Laboratory	#000651
OT-II (B6.Cg-Tg^*(TcraTcrb)425Cbn*^/J)	Jackson Laboratory	#004194
NLRP3^-/-^ (B6.129S6-*Nlrp3*^*tm1Bhk*^/J)	Jackson Laboratory	#021302

**Software and algorithms**		

LSRFortessa HTS with FACS Diva	BD Biosciences	v8.0.2
FlowJo	BD Biosciences	v10
Graphpad Prism	Graphpad	v10
Affinity Designer	Affinity Designer	V1.10.8
Microsoft Word	Microsoft	
Microsof Excel	Microsoft	
Fluostar Omega	BMG Labtech	

### Method details

For the study, 8–10-week-old males and females C57BL/6J (H-2^b^), Balb/C (H-2^d^), OT-II (B6.Cg-Tg^*(TcraTcrb)425Cbn*^/J), and NLRP3^-/-^ (B6.129S6-*Nlrp3*^*tm1Bhk*^/J) mice were purchased from Jackson Laboratory. Mice and littermate controls were randomly assigned to the treated and control groups at the start (the sample size for each group in each experiment was 5). Mice were housed under controlled conditions (22 ± 2C) with a 12-hour light/dark cycle and had *ad libitum* access to food and water. All animals were kept under a specific pathogen-free facility at Boston Children’s Hospital. All mouse experimental protocols were approved by the Institutional Animal Care and Use Committees of Boston Children’s Hospital under protocol number 20-01-4117/00001847.

Human PBMC samples (buffy coat donations) were collected following written informed consent from each donor. The protocols for sample collection were approved in advance by the local medical ethics committee (IRB-P00038916), in accordance with the Declaration of Helsinki.

#### Drug administration

For adjuvant administration, mice received either saline or alum (8mg in 200 μL) (Thermo Fischer Scientific) intraperitoneally every other day three times. For IL-1 signaling blockade, KINERET® (Anakinra) was injected intraperitoneally at a dose of 30mg/kg at time points described in each figure.

#### Preparation of single-cell suspensions

The mice were euthanized by CO_2_ and spleens were aseptically removed, minced, filtered through a sterile 70 μm filter (BD, USA), and centrifuged to collect a single-cell suspension. Splenocytes underwent red blood cell lysis with RBC lysis using ACK lysis buffer (ThermoFisher Scientific). Briefly, the cell suspension was centrifuged at 300 × g for 5 minutes at 4°C, and the supernatant was discarded. The pellet was resuspended in freshly prepared RBC lysis buffer (0.15 M ammonium chloride, 10 mM potassium bicarbonate, 0.1 mM EDTA, pH 7.2–7.4) and incubated at room temperature for 5 minutes with occasional gentle mixing. Following incubation, the reaction was quenched by adding an excess volume of PBS or culture medium, and the cells were centrifuged at 300 × g for 5 minutes. The supernatant containing lysed RBCs was removed, and the cell pellet was washed twice with PBS before resuspending in the appropriate medium for downstream applications.

#### Cell culture

Complete RPMI-1640 medium was prepared by supplementing RPMI-1640 basal medium (Corning) with 10% heat-inactivated fetal bovine serum (FBS) (Gibco), 2 mM L-glutamine (Gibco), 100 U/mL penicillin (Gibco), and 100 μg/mL streptomycin (Gibco). The medium was filtered through a 0.22 μm filter for sterility and stored at 4°C until use. Before cell culture, the medium was warmed to 37°C in a water bath.

#### OVA immunization protocol

Wild-type or NLRP3 deficient mice were adoptively transferred with 5x10^6^ total splenocytes from OT-II mice intravenously. 24h after the adoptive transfer, mice were immunized subcutaneously with OVA grade-V (100μg) (Sigma-Aldrich) adsorbed either to Alum Imject (1.5mg), or to Resiquimod (R848) (InvivoGen) (50μg) in a homologous prime and boost (day 0 and 7) protocol. At day 15 after priming, mice were euthanized, and spleens were collected for phenotyping and restimulation *in vitro* with 2μg/mL of OVAp 323-339 (InvivoGen), and blood was collected for serum OVA-antibody detection. For MDSCs adoptive transfer experiments, 2x10^6^ of spleen-isolated CD11^+^GR1^+^ were injected intravenously 24h before the OVA immunization protocol.

#### Enzyme-linked immunosorbent assay (ELISA)

Blood samples were obtained from mice via cardiac punction he collected blood samples were left to sit overnight at 4°C, followed by centrifugation the next day to obtain serum samples which were then frozen at −20°C. OVA-specific IgG levels were measured using an indirect ELISA. Briefly, 96-well EIA/RIA plates (Corning) were coated overnight at 4°C with 100 μL of OVA (50 μg/mL) in carbonate-bicarbonate buffer (pH 9.6). The plates were then washed three times with PBS-Tween (0.05% Tween-20 in PBS) and blocked with 5% bovine serum albumin (BSA) at room temperature for 1 hour to prevent nonspecific binding. After blocking, serum samples were diluted in PBS and added to the wells followed by incubation for 2 hours at room temperature. The plates were then washed three times with PBS-Tween, and HRP-conjugated goat anti-mouse IgG (Sigma Aldrich) was added and incubated for 1 hour. After another series of three washed with PBS-Tween, the reaction was developed using OPD substrate (o-phenylenediamine dihydrochloride) (Thermo Fisher Scientific) and stopped with 2N sulfuric acid. Absorbance was measured at 450 nm using a microplate reader, and IgG titer was defined as the highest serum dilution yielding an optical density (OD) value exceeding the mean OD of negative control samples plus two standard deviations.

#### MDSCs enrichment

CD11b^+^GR1^+^ cells were isolated using negative selection from mouse spleens using the EasySep mouse MDSC (CD11b^+^Gr1^+^) isolation kit (StemCell) according to the manufacturer’s protocol. Purity of isolated MDSCs were verified using flow cytometry to be >90%. Isolated MDSCs were cultured in RPMI 1640 medium (ThermoFisher Scientific) containing 10% fetal bovine serum (Corning), 200μg/mL penicillin (ThermoFisher Scientific), 200 U/mL streptomycin (ThermoFisher Scientific), and 0.05mM 2-mecaptoethanol (Sigma-Aldrich) for 24h in the presence or not of LPS (200ng/mL) (Sigma-Aldrich cat 297-473-0).

#### Flow cytometry

The following antibodies were used for surface staining: CD11b (clone: M1/70; Invitrogen), Ly6C (clone: HK1.4; BioLegend), Ly6G (clone: 1A8; BioLegend), CD3 (clone: 17A2; Invitrogen), CD4 (clone: GK1.5; Invitrogen), TCR Va2 (clone B20.1, Biolegend). program cell death protein ligand 1 (PD-L1; clone: 10F.9G2; BioLegend). Samples were fixed and permeabilized with Fix/Perm buffer according to the manufacturer’s instruction (eBioscience) before intracellular protein staining. The following antibodies were used for intracellular staining: Tbet (clone: 4B10; BioLegend), GATA3 (clone: 16E10A23; BioLegend), RORγT (clone: Q31-378; BioLegend), Foxp3 (clone: MF-14; BioLegend), IFN-γ (clone: AN-18; BioLegend), IL-4 (clone: 11B11; BioLegend), IL-17A (clone: TC11-18H10.1; BioLegend), LAP (clone: TW7-16B4; BioLegend). Splenocytes were cultured with GolgiPlug (BD Biosciences) for 48h for intracellular cytokine detection. Cell viability was determined Fixable Viability Dye eFluor™ 780 (eBioscience™), Zombie Red™ or Zombie Aqua™ Fixable Viability (Biolegend). Samples were collected on LSRFortessa HTS with BD FACSDiva v8.0.2 software (BD Biosciences). FlowJo v10 was used for flow data analysis.

#### CD4 T cell suppression assay

Lymphocyte proliferation was assessed using CellTrace™ Violet (CTV) dye dilution. Briefly, naïve CD4 T cells isolated from the spleen of OT-II mice were labeled with 5 μM CTV (Thermo Fischer Scientific) by incubating at 37°C for 20 minutes in the dark. The labeling reaction was quenched by adding complete culture medium supplemented with 10% fetal bovine serum (FBS), followed by washing with fresh medium. Labeled cells were then added to culture with 10^5^ C57BL/6 total splenocytes and stimulated with 1μg/mL of OVAp 323-339 (InvivoGen) and cultured at 37°C for 72–96 hours. CD11b^+^GR1^+^ cells were isolated from alum-treated or saline-treated animals and cultured in different MDSC to T cell ratios (1:1, 1:4, and 1:8) to assess MDSCs suppressor function. After incubation, cells were harvested and analyzed by flow cytometry. CTV fluorescence intensity was measured in the appropriate channel, with progressive dye dilution indicating successive cell divisions. Data were analyzed using FlowJo software to determine the proliferation index and percentage of dividing cells.

#### Western blotting

CD11b^+^GR1^+^ cells were isolated using negative selection from mouse spleens using the EasySep mouse MDSC (CD11b^+^Gr1^+^) isolation kit (StemCell) according to the manufacturer’s protocol and resuspended in RIPA lysis buffer (Thermo Fischer Scientific) containing protease and phosphatase inhibitors (Thermo Fischer Scientific). Total protein was assessed by BCA assay (Thermo Fischer Scientific). 20μg of total protein was mixed with loading buffer, boiled for 5 minutes at 90°C, and cooled to room temperature. SDS-PAGE gel electrophoresis was performed in NuPAGE 12% acrylamide gels submerged in tris buffer pH 7.4. Proteins were separated at 70V for 10 minutes, followed by 60 mins at 120V. The gel was assembled into a western blot sandwich per manufacturers’ instructions (BioRad), and the MW ladder and samples were transferred to a 0.45um PVDF membrane using a TransBlot Turbo transfer machine (BioRad) at 25V for 7 mins. The resulting western blot was washed in tris buffer and blocked in 3% BSA for 1 hour, followed by overnight incubation with primary mouse anti-IDO1 (clone: 2E2/IDO1; BioLegend). The blot was washed with Tris and incubated with secondary goat anti-mouse-HRP (A9044, Sigma Aldrich) for 2 hours at room temperature. The blot was washed in tris and ECL cocktail (BioRad), applied per the manufacturers’ instructions, and exposed accumulatively for a maximum of five minutes on a Biorad ChemiDoc for 5 minutes. Images were processed using ImageLab (BioRad).

#### Human PBMC isolation for stimulation *in vitro*

All buffy coat donations were collected following written informed consent from each donor. The protocols for sample collection were approved in advance by the local medical ethics committee (IRB-P00038916), in accordance with the Declaration of Helsinki. PBMCs were isolated using Lymphoprep density gradient medium centrifugation (Stemcell Technologies). Blood was diluted in a 1:1 ratio with PBS 1x, 2% fetal bovine serum, and 4 mM EDTA to prevent cell death and clumping. The diluted blood was carefully layered on 10 mL of Lymphoprep medium at room temperature in a 50 mL Falcon tube, avoiding mixing. The tube was then placed in a centrifuge and spun for 30 minutes at 900xg at 23°C with minimum deceleration. After centrifugation, the cell ring between the upper layer (plasma) and the lower layer (Lymphoprep) was collected and washed with PBS 1x, 2% fetal bovine serum, and 4 mM EDTA. Red blood cells were lysed using 10 ml of ACK lysing buffer (Gibco) for 10 minutes at room temperature. The PBMCs were washed again, resuspended in PBS 1x, 2% fetal bovine serum, and 4 mM EDTA, counted, and kept on ice for further use. Isolated PBMCs were plated 3x10^5^ in each well of a round bottom 96-well plate and stimulated with Alum Imject™ at 250 or 500μg/mL for 24h for one, two or three times, and later stimulated with LPS (200ng/mL) for an additional 24h, all in 200μL/well. In between alum stimulations the supernatant was changed, stored at -80°C, and fresh complete RPMI was added. Cell viability after alum stimulation was assessed by LDH release, measured using CyQUANT™ LDH Cytotoxicity Assay (ThermoFisher Scientific). In order to calculate the viability, we used controls of 3x10^5^ cells cultured in complete RPMI for 0% cell death, and cells cultured in complete RPMI, but in the last 30 minutes of culture, the media was replaced by complete RPMI containing Triton X-100 0.1%(Merck) for 100% cell death. The activity of LDH was measured in the supernatant of the cell cultures according to manufacturer’s instructions. Cell death was calculated as a percentage of the 100% cell death control.

#### Cytokine detection

Cytokine release in the supernatant was assessed using LEGENDplex™ Mouse Inflammation Panel 1 (740150; BioLegend) and LEGENDplex™ Human Inflammation Panel 1 Standard (740811; BioLegend). Serum cytokines were assessed by Mouse IL-1 beta/IL-1F2 DuoSet ELISA (DY40105; R&D Systems™) and Mouse IL-1 alpha/IL-1F1 Quantikine ELISA Kit (MLA00; R&D Systems™).

#### Nitric oxide detection

Nitric oxide production by isolated CD11b^+^Gr1^+^ cells cultured *in vitro* with or without LPS stimulation (200ng/mL) was assessed by Griess Reagent Kit (Invitrogen), using the manufacturer instructions. Nitric oxide (NO) production was indirectly measured by quantifying nitrite NO_2_ levels in cell culture supernatants using the Griess reaction. Briefly, 100 μL of culture supernatant was mixed with an equal volume of Griess reagent (1% sulfanilamide in 5% phosphoric acid and 0.1% N-(1-naphthyl)ethylenediamine dihydrochloride) in a 96-well microplate. The reaction was incubated at room temperature for 20 minutes in the dark, allowing color development. Absorbance was measured at 540 nm using a microplate reader. A sodium nitrite NaNO_2_ standard curve (0–100 μM) was prepared in parallel to determine nitrite concentrations in the samples. The results were expressed as μM nitrite, based on interpolation from the standard curve.

#### Allogeneic pancreatic islet transplantation

Diabetes was induced in recipient mice (wild-type or NLRP3^-/-^) by intraperitoneal injection of streptozotocin (STZ) at a dose of 240 mg/kg (Sigma-Aldrich). Mice with blood glucose levels exceeding (e.g., 300 mg/dL for two consecutive days) were considered diabetic and selected for transplantation. Donor pancreatic islets were isolated from 8-week-old Balb/c mice using collagenase digestion and density gradient centrifugation. Briefly, the pancreas was perfused with collagenase solution (Roche), digested at (37°C for 15 minutes), and purified by density gradient centrifugation. Isolated islets were handpicked under a stereomicroscope and cultured in complete RPMI-1640 medium at 37°C with 5% CO_2_ before transplantation. For transplantation, 300–500 purified islets were implanted under the kidney capsule of recipient diabetic mice. Mice were anesthetized using isoflurane and a small incision was made to expose the kidney. Islets were loaded into a Hamilton syringe and gently injected beneath the kidney capsule. The incision was closed using absorbable sutures or wound clips, and mice were monitored postoperatively. Blood glucose levels were measured daily to assess graft function. Mice with sustained normoglycemia (e.g., blood glucose <200 mg/dL) were considered successfully engrafted.

#### Allogeneic cutaneous hypersensitivity

To assess allogeneic cutaneous hypersensitivity, C57BL/6 wild-type and NLRP3^-/-^ mice were immunized subcutaneously with 20x10^6^ Balb/c splenocytes in 100 μL PB at the dorsal flank. Seven days post-immunization, mice were challenged with 20x10^6^ Balb/c splenocytes injected in 10 μL PBS into the base of the ear pinna. Ear swelling was measured using a digital caliper before and 2-, 4- and 24-hours after challenge to quantify the delayed-type hypersensitivity (DTH) response. The degree of swelling was calculated as the difference in ear thickness between pre- and post-challenge measurements. Mice injected with saline alone served as negative controls.

### Quantification and statistical analysis

All experiments were repeated at least three times with a minimum number of 5 animals per group (n=5). Statistical analysis was performed using GraphPad Prism version 10. Two-tailed unpaired Student t test was used to calculate differences between experimental animals. One-way analysis of variance was used for multiple comparisons. Graft survival significance was assessed by Kaplan-Meier/Mantel-Cox log-rank test. P value of <0.05 was considered a statistically significant difference.
