# Supplementary material for: Adjuvant conditioning shapes the adaptive immune response promoting immunotolerance via NLRP3/interleukin-1
Source: iScience. 2025 May 13;28(6):112653. doi: 10.1016/j.isci.2025.112653 (PMC12173022; doi:10.1016/j.isci.2025.112653)

iScience, Volume *28*

Supplemental information

Adjuvant conditioning shapes the adaptive immune response promoting immunotolerance via NLRP3/interleukin-1

Thais Boccia, Weikang Pan, Victor Fattori, Rodrigo Cervantes-Diaz, Michael S. Rogers, Ivan Zanoni, and Alex G. Cuenca

Supplemental information

Document S1.

Figure S1: Flow cytometry gating strategies and immunization controls.

Figure S2: IL-1 production in vivo after adjuvant conditioning and immunization controls.

Figure S3: MDSC expansion after adjuvant conditioning and T cell proliferation controls.

Figure S4: Characterization of human PBMCs after adjuvant conditioning in vitro.

Figure S5: Cytokine production by human PBMCs after adjuvant conditioning in vitro.

Supplemental figure legends

Supplemental Figure 1: Flow cytometry gating strategies and immunization controls. **A:** Gating strategy to find the population of OVA-specific CD4 T cells in the spleen of immunized mice. **B:** Total number of OVA-specific Th1, Th2, Th17 and Tregs cells, evidenced by the expression of $CD4^+V\alpha^+Tbet^+$, $CD4^+V\alpha^+GATA3^+$, $CD4^+V\alpha^+ROR\gamma T^+$, and $CD4^+V\alpha^+Foxp3^{+,}$ respectively, in splenocytes from C57BL/6 mice treated with either saline or alum before the immunization with OVA-Alum or OVA alone. **C:** Total number of OVA-specific Th1, Th2, Th17 and Tregs, evidenced by the expression of $CD4^+V\alpha^+IFN\text{-}\gamma^+$, $CD4^+V\alpha^+IL\text{-}4^+$, $CD4^+V\alpha^+IL\text{-}17A^+$ and $CD4^+V\alpha^+LAP^+$ in splenocytes from C57BL/6 mice treated with either saline or alum before the immunization with OVA-Alum or OVA alone, and further cultured with OVAp (2μg/mL) *in vitro* for 48h in the presence of brefeldin-A. Data shown represent three or more experiments, and are expressed as mean±SEM; Student t-test was used for analysis; P values are indicated in the graphs. All statistical analyses were performed using GraphPad Prism™.

Supplemental Figure 2: IL-1 production in vivo after adjuvant conditioning and immunization controls. **A:** Serum levels of IL-1α and IL-1β 24h after the last i.p. injection of Alum Imject™ in

C57BL/6 and NLRP3 deficient mice. **B:** Total number of OVA-specific CD4 T cells and serum titer of OVA-specific IgE **(C)** in C57BL/6 and NLRP3 deficient mice treated with either saline or alum before the immunization with OVA-RSQ. **D:** Total number of OVA-specific Th1, Th2, Th17 and Tregs cells, evidenced by the expression of $CD4^+V\alpha^+Tbet^+$, $CD4^+V\alpha^+GATA3^+$, $CD4^+V\alpha^+ROR\gamma T^+$, and $CD4^+V\alpha^+Foxp3^+$ respectively in splenocytes from C57BL/6 and NLRP3 deficient mice treated with either saline or alum before the immunization with OVA-RSQ, and total number of OVA-specific Th1, Th2, Th17 and Tregs, evidenced by the expression of $CD4^+V\alpha^+IFN\text{-}\gamma^+$, $CD4^+V\alpha^+IL\text{-}4^+$, $CD4^+V\alpha^+IL\text{-}17A^+$ and $CD4^+V\alpha^+LAP^+$ in splenocytes from C57BL/6 and NLRP3 deficient mice treated with either saline or alum before the immunization with OVA-RSQ, and further cultured with OVAp (2μg/mL) *in vitro* for 48h in the presence of brefeldin-A. **E:** Total number of OVA-specific Th1, Th2, Th17 and Tregs cells, evidenced by the expression of $CD4^+V\alpha^+Tbet^+$, $CD4^+V\alpha^+GATA3^+$, $CD4^+V\alpha^+ROR\gamma T^+$, and $CD4^+V\alpha^+Foxp3^+$ respectively in splenocytes from C57BL/6 mice treated with either saline, alum or alum $^+$ anakinra (AC$^+$AK) before the immunization with OVA-Alum. **F:** Serum titer of OVA-specific IgE in C57BL/6 mice treated with either saline, alum or alum $^+$ anakinra (AC$^+$AK) before the immunization with OVA-Alum.

Supplemental Figure 3: MDSC expansion after adjuvant conditioning and T cell proliferation controls. **A:** Total cell count in spleens from mice treated with either saline or alum 24h after the last injection of Alum Imject^{TM} in C57BL/6 mice. **B:** Spleen weight from mice treated with either saline or alum 24h after the last injection of Alum Imject^{TM} in C57BL/6 mice. **C:** Total count of $CD11b^+Ly6C^+$ and **(D)** $CD11b^+Ly6G^+$ cells in spleens from mice treated with either saline or alum 24h after the last injection of Alum Imject^{TM} in C57BL/6 mice. **E:** Image of spleens from mice treated with either saline or alum 24h after the last injection of Alum Imject^{TM} in C57BL/6 mice. **F:** Image of spleens from C57BL/6 mice treated with either saline, alum or alum + anakinra (AC+AK) 24h after the last injection of Alum Imject^{TM}. **G:** Histograms showing PDL-1 expression in

$CD11b^+Ly6C^+$ and $CD11b^+Ly6G^+$ cells in spleens from mice treated with either saline or alum 1 day, 7 days, or 14 days after the last injection of Alum Imject^{TM}. **H:** PDL-1 expression shown by MFI. **I:** IDO expression in isolated $CD11b^+GR1^+$ cells from spleens from mice treated with either saline or alum 1 day, 7 days, or 14 days after the last injection of Alum Imject^{TM}. **J:** Percentage of $CD4^+V\alpha^+CTV^{low}$ cells in a suppression assay using isolated $CD11b^+GR1^+$ from saline or alum-treated C57BL/6 and NLRP3 deficient mice. **K:** Percentage of $CD4^+V\alpha^+FVD^+$ cells in a suppression assay using isolated $CD11b^+GR1^+$ cells from saline or alum-treated C57BL/6 and NLRP3 deficient mice. **L:** Percentage of $CD4^+V\alpha^+CTV^{low}$ cells in a suppression assay using isolated $CD11b^+GR1^+$ from saline, alum or alum + anakinra (AC+AK) C57BL/6 mice. **M:** Percentage of $CD4^+V\alpha^+FVD^+$ cells in a suppression assay using isolated $CD11b^+GR1^+$ cells from saline, alum, or alum + anakinra (AC+AK) C57BL/6 mice.

Supplemental Figure 4: Characterization of human PBMCs after adjuvant conditioning in vitro. PBMCs were plated $3x10^5$ in each well of a round-bottom 96-well plate and stimulated with Alum Imject^{TM} at 250 or 500μg/mL for 24h for one, two, or three times. In between alum stimulations, the supernatant was changed, stored at -80 °C, fresh complete RPMI was added, and cells were collected for flow cytometry phenotyping. **A:** Percentage and total cell count of viable monocytes, B cells and T cells after each alum treatment, assessed by flow cytometry, and viability dye staining of $CD66b^{neg}$ $CD11b^+CD14^+$ cells (monocytes), $CD66b^{neg}$ $CD19^+$ (B cells), and $CD66b^{neg}$ $CD3^+$ (T cells). **B:** Flow cytometry phenotyping of PBMCs stimulated with Alum Imject^{TM} at 250 or 500μg/mL for 24h for one, two, or three times.

Supplemental Figure 5: Cytokine production by human PBMCs after adjuvant conditioning in vitro. $3x10^5$ total PBMCs from five different healthy donors were stimulated with alum (**A:** 250μg/mL or **B:** 500μg/mL) once, twice, or three times for 24h in round-bottom 96-well plates, in complete RPMI

(described in Star Methods). The supernatant was collected for cytokine measurement using LEGENDplex™ Human Inflammation Panel 1. IL-1β, IFN-γ TNF-α, IL-6, IL-8, IL-10 and IL-12 production by PBMCs stimulated with alum (250μg/mL) once, twice or three times. Numbers in squares show the median of the 5 donor cells for each cytokine.

Supplemental Figure 1

A

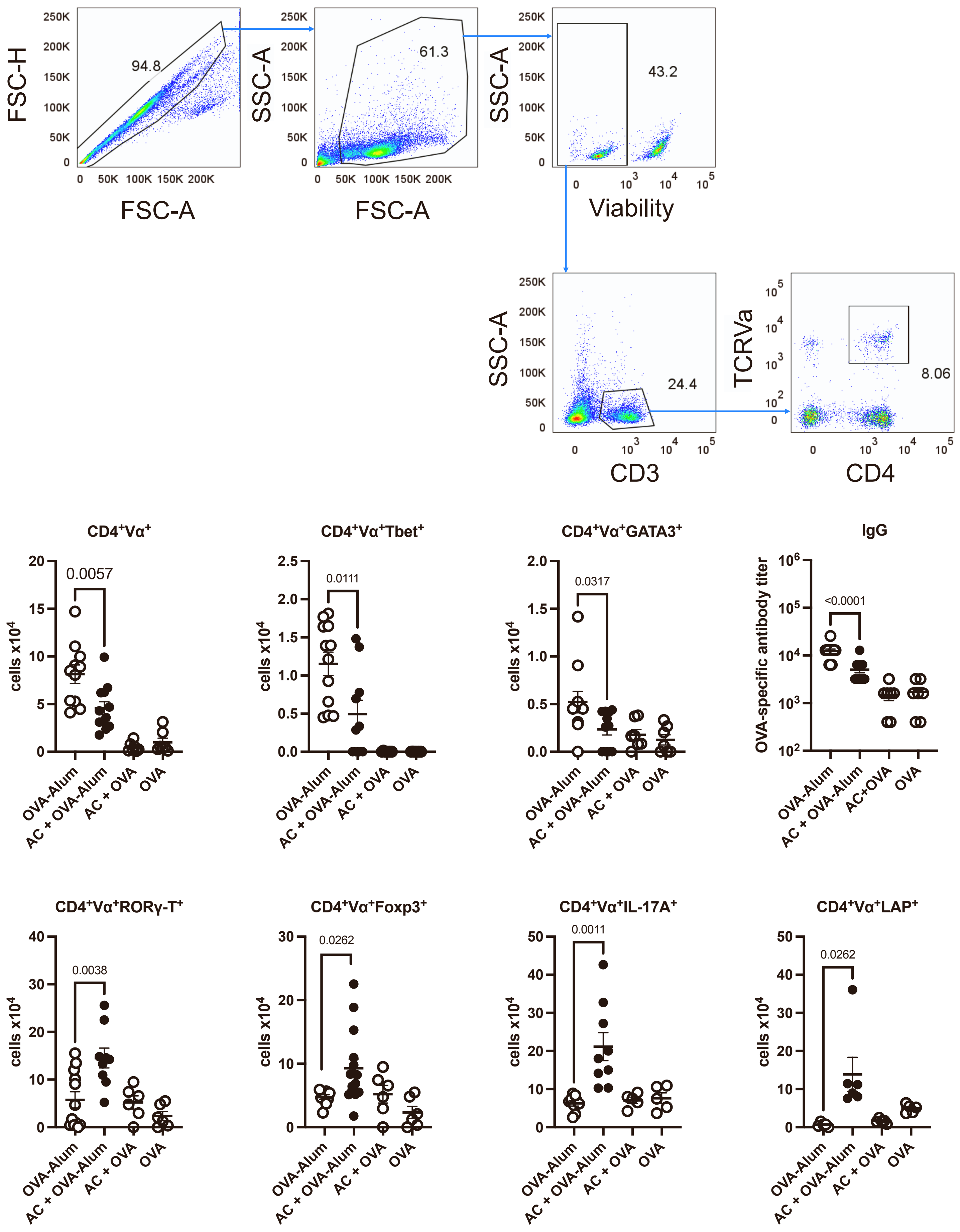

Supplemental Figure 2

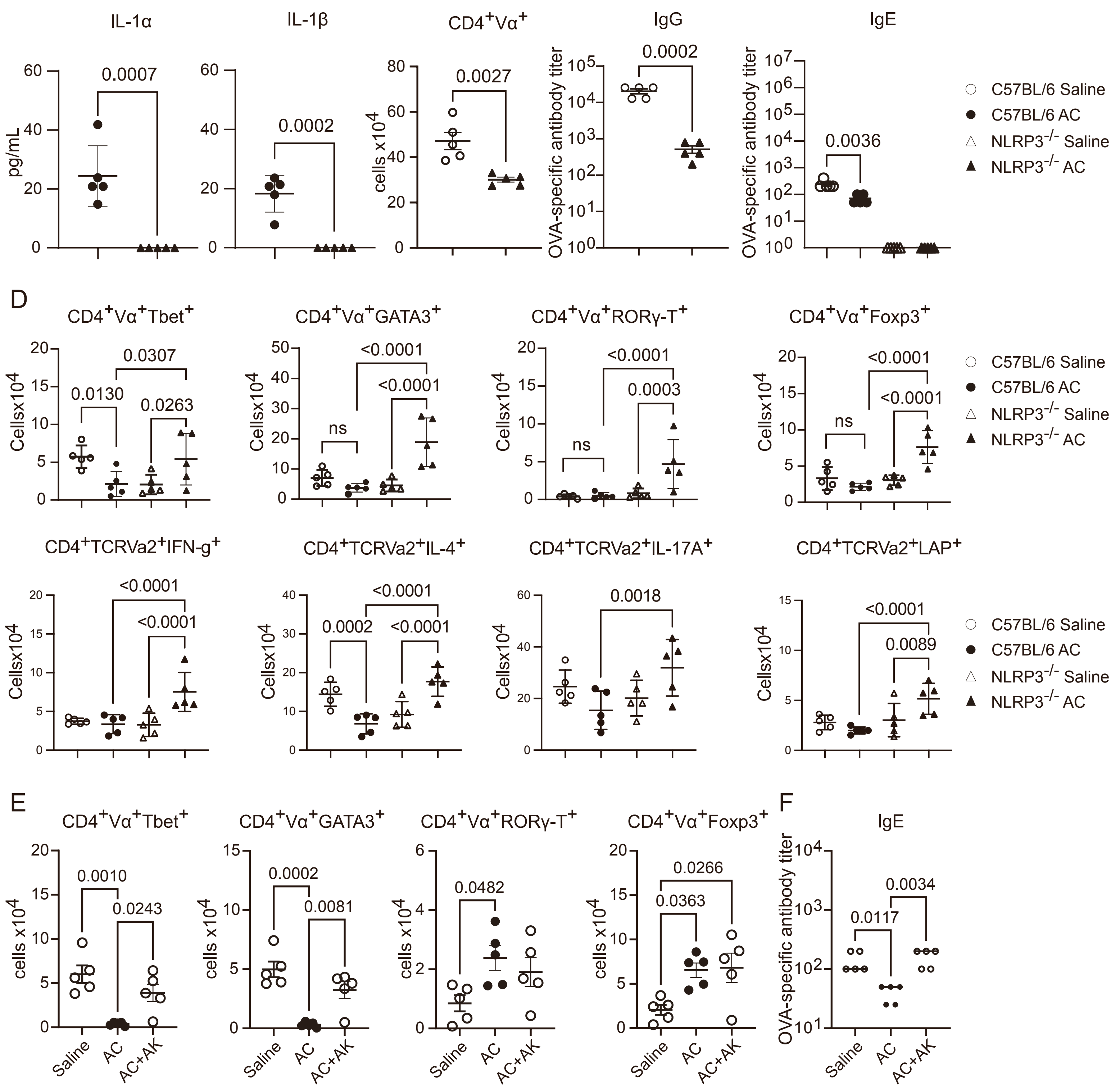

Supplemental Figure 3

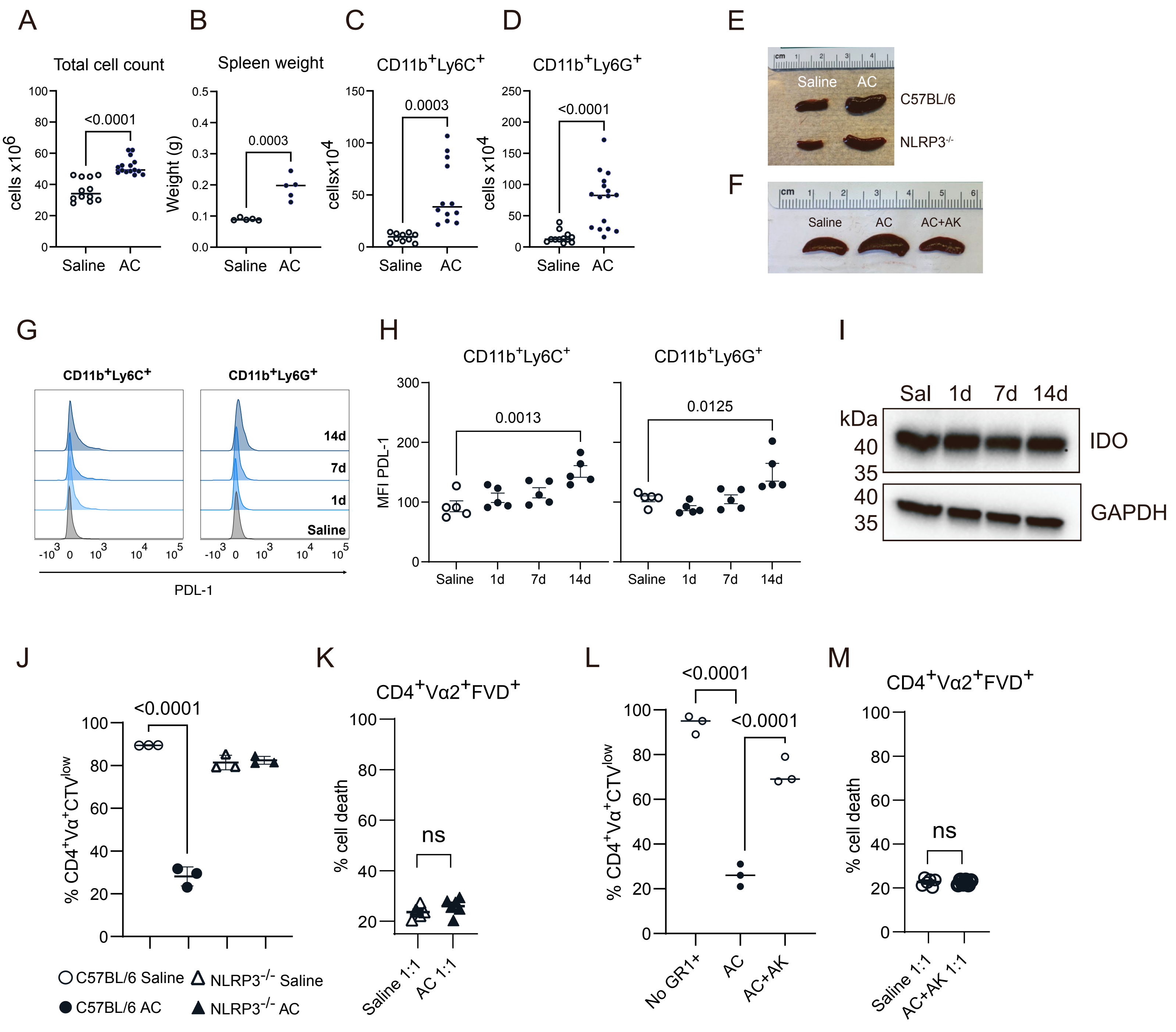

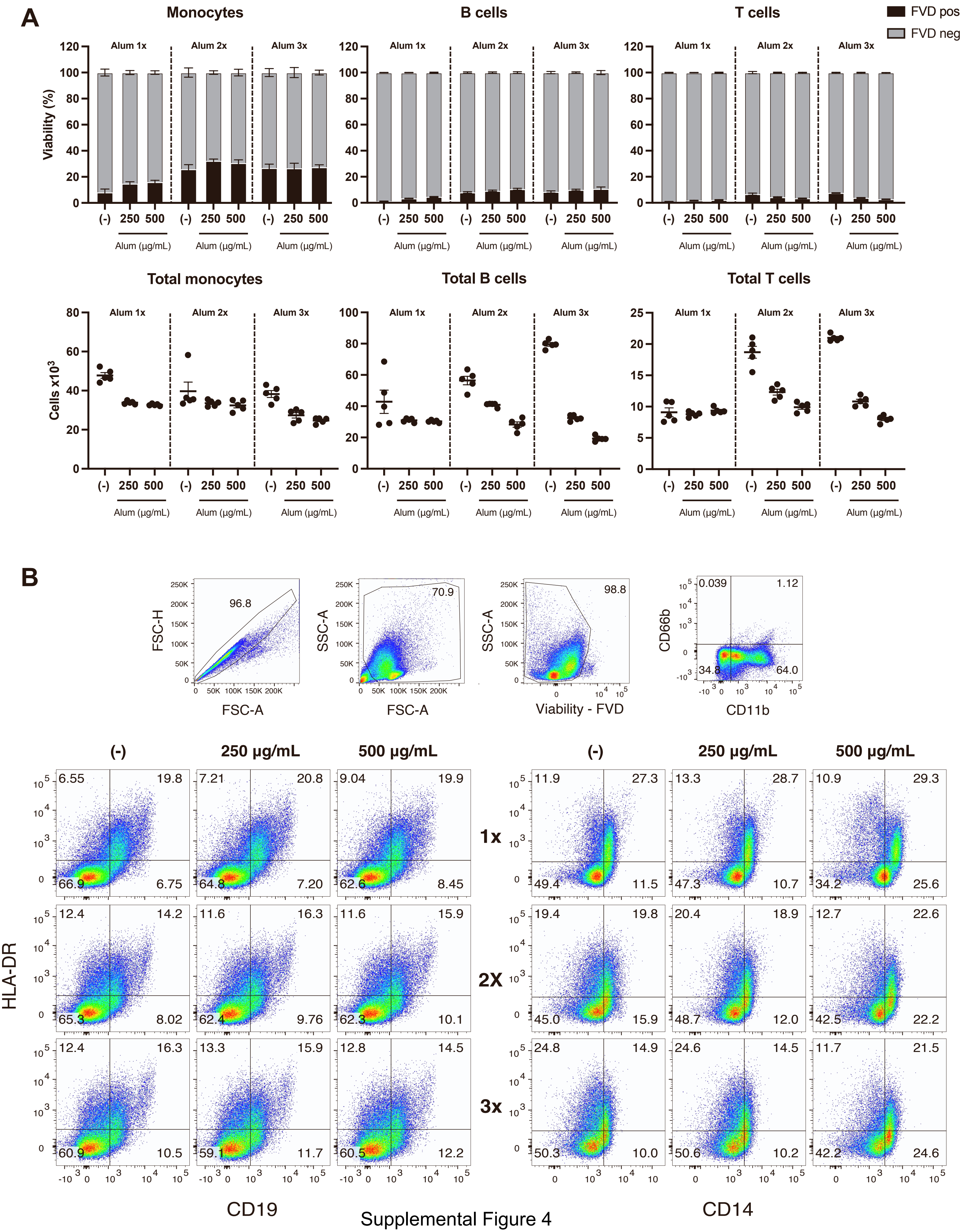

Supplemental Figure 4

Supplemental Figure 5

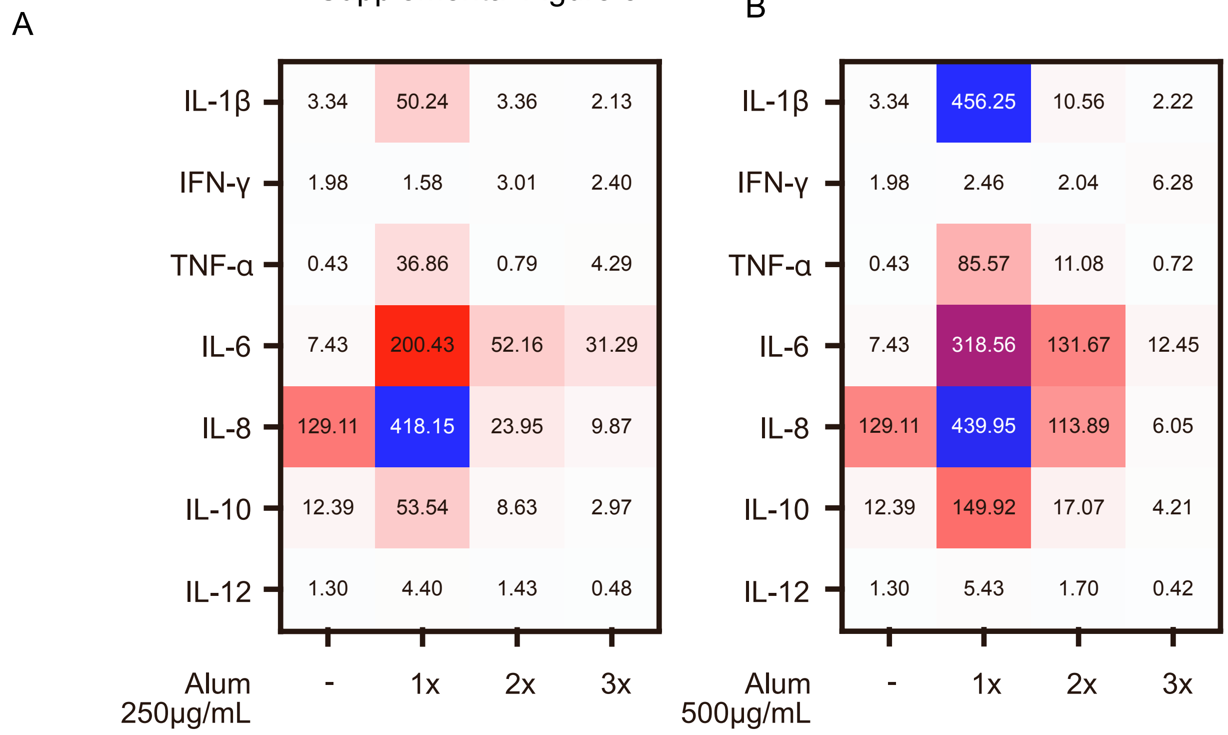

Supplement: Document S1. Figures S1–S5 [file mmc1.pdf]
